# Biomarkers for complex post-traumatic stress disorder: translational and evolutionary perspectives

**DOI:** 10.3389/fpsyt.2026.1786811

**Published:** 2026-04-07

**Authors:** Ravi Philip Rajkumar

**Affiliations:** Department of Psychiatry, Jawaharlal Institute of Postgraduate Medical Education and Research, Pondicherry, India

**Keywords:** animal models, appeasement displays, complex PTSD, evolutionary theory, oxytocin, post-traumatic stress disorder, serotonin, vasopressin

## Introduction

Post-traumatic stress disorder (PTSD) is a chronic mental illness that occurs following exposure to traumatic stressors such as combat, disasters, or assault. It is characterized by a triad of re-experiencing of the trauma, avoidance of triggers for such recollections, and increased vigilance towards threats (1). In 1992, Judith Herman described a variant of PTSD that occurred in persons who had undergone prolonged or repeated traumatic stress, such as hostages, prisoners of war, concentration camp survivors, or victims of chronic familial abuse or violence. Apart from the classical “triad” seen in PTSD, these patients experienced somatic, dissociative, and mood symptoms, alterations in identity, and disturbed interpersonal relationships. She proposed the term “complex PTSD” to describe such cases (2).

A syndrome akin to “complex PTSD” was proposed for inclusion in the fourth edition of the Diagnostic and Statistical Manual of Mental Disorders (DSM-IV) under the name “Disorders of extreme stress, not otherwise specified” (DES-NOS). The alterations in mood, identity and relationships described by Herman were also included in the tenth edition of the World Health Organization (WHO)’s International Classification of Diseases and Disorders (ICD-10) with the label “Enduring personality change after catastrophic experience (F62.0).” However, DES-NOS was not included in the final version of DSM-IV, and ICD-10 category F62.0 was rarely used in practice (3). Based on research over the next two decades, the concept of complex PTSD was refined and validated in diverse settings. Complex PTSD (C-PTSD) was redefined as a syndrome consisting of both the PTSD triad, and a second triad of disturbances in self-organization (DSO), characterized by disturbances of mood (numbing or increased reactivity), difficulties in interpersonal relationships, and a negative self-image. Such symptoms were defined as occurring in the context of complex trauma – that is, trauma which is repeated or prolonged. This definition of complex PTSD has been included in the most recent WHO classification of mental disorders (ICD-11). It is estimated that about 2-8% of the world’s population suffers from C-PTSD, with much higher rates observed in vulnerable groups such as refugees and survivors of childhood abuse (4–6).

Optimal treatment strategies for C-PTSD are still in development. Pharmacological treatments for PTSD may improve symptoms in the PTSD triad, but do not have proven benefits for DSO. Trauma-focused psychotherapies improve PTSD triad symptoms, depression, anxiety, and insomnia, but their effect on overall quality of life – a measure of DSO – is low (7). There are also significant variations in efficacy between psychotherapies based on different theoretical models and techniques (8). Novel therapies such ketamine and psilocybin have been suggested as alternatives, but though they have some benefits in PTSD, their efficacy in C-PTSD has not been evaluated (9, 10). The development of more effective treatments for this chronic and disabling condition would require a better understanding of the neurobiology of C-PTSD, particularly in relation to symptoms of DSO, which do not appear responsive to currently available treatments (11).

## What is known about the neurobiology of complex PTSD?

The past four years have seen remarkable advances in our understanding of the pathophysiology of PTSD. Initial work focused on dysregulation of monoamine neurotransmitters such as serotonin (5-HT, and on altered functioning of the hypothalamic-pituitary-adrenal (HPA) axis (12). It is now known that a host of complex physiological and biological alterations occur in PTSD, including alterations in glutamatergic and peptidergic transmission, increased oxidative stress, immune-inflammatory dysregulation, and accelerated cellular aging. These changes appear to arise from an interaction between genetic variants influencing these pathways, “sensitizing” experiences such as childhood adversity, and exposure to one or more traumatic stressors (5, 13, 14). These far-reaching systemic changes explain the strong associations between PTSD and other medical and neurological disorders (15).

Relatively less is known about the biological substrates of C-PTSD. It can be assumed that the “classical” symptoms of PTSD seen in these patients have the same putative origin as those of non-complex PTSD, and many early studies on the biology of PTSD included patients with undiagnosed C-PTSD, such as veterans or refugees. In contrast, little is known about the neural or biochemical bases for DSO (16). Experts in the field agree that comprehensive research is warranted, and certain important initiatives, such as a biobank aimed at comparing PTSD and C-PTSD, have already been launched (17).

Dissociative symptoms, characterized by disturbances in identity or in the integration of psychological functions, are commonly seen in C-PTSD, and may be correlated with DSO. A candidate gene study found an association between dissociative symptoms in PTSD and the *FKBP5* gene, involved in regulating glucocorticoid receptor responsiveness to stress, but none of the patients in this study was specifically evaluated for C-PTSD (18). More promisingly, a genome-wide association study (GWAS) found possible associations between “dissociative PTSD” – a condition similar to C-PTSD – and the genes encoding adenylyl cyclase 8 *(ADCY8)* and dipeptidyl-peptidase 6 *(DPP6)*. The former gene has been associated with fear-based memories and synaptic plasticity, while the latter is linked to synaptic integrity. Variants in these genes may confer vulnerability to C-PTSD, but this hypothesis requires formal verification (19).

## Are there reliable biomarkers of C-PTSD?

Potential biomarkers of PTSD, prior to the inclusion of C-PTSD in psychiatric nosology, have been studied extensively. Structural and functional imaging has revealed altered functioning of the brain’s default mode, salience, and central executive networks in patients with PTSD (20). Studies of peripheral blood markers have found consistent evidence of low baseline cortisol, elevated levels of pro-inflammatory cytokines such as interleukin-1 and tumor necrosis factor alpha, reduced antioxidant activity, and elevated indices of the metabolic syndrome (21, 22). More recently, alterations in markers of energy metabolism, such as arginine and pyruvic acid, have also been identified in this patient group (23). Though this research has shed valuable light on the pathophysiology of PTSD, no specific marker has yet proved sensitive or specific enough to aid diagnosis or treatment of this disorder. Moreover, there is substantial variability across studies (21).

In contrast to this, there is relatively little research on biomarkers specifically associated with C-PTSD. Neuroimaging has been the most frequently used method in this search to date. Structurally, patients with C-PTSD related to childhood abuse appear to have reduced volumes of the right anterior cingulate cortex (ACC), orbitofrontal cortex (OFC), and bilateral hippocampus and amygdala (24, 25). Functionally, C-PTSD is associated with increased activation of the left anterior cingulate cortex (ACC), hippocampus, and parahippocampal gyrus when patients are asked to learn and recall negative words (such as “panic” or “rape”) (26, 27). A similar increase in activation of the ACC, dorsolateral prefrontal cortex (dlPFC) and ventromedial prefrontal cortex (vmPFC) was seen when persons with C-PTSD were exposed to trauma-related words (28). Alterations in the connectivity of the default mode network (DMN) have also been reported, but these appear to be common to PTSD and C-PTSD (29). Overall, there is evidence of reduced volume and increased activation of cognitive structures that may reduce limbic overactivity in relation to traumatic cues in C-PTSD. Similar structural and functional changes can be seen in PTSD (30), and there is no evidence that the above findings correlate with symptoms specific to C-PTSD, particularly DSO. Therefore, it is unclear whether these changes, though significant, are specific or sensitive enough to constitute biomarkers for C-PTSD.

Physiological studies, though less numerous, have also identified anomalies associated with C-PTSD. Electroencephalographic (EEG) analysis of patients acutely ill with C-PTSD has revealed reduced functional connectivity in the default mode network (DMN) and in regions connected to the prefrontal cortex and ACC. These changes normalized after in-patient treatment with a focus on trauma-oriented psychotherapy (31). When adolescents with C-PTSD or PTSD underwent a structured interview about their traumatic experiences, both groups had elevated heart rates during the interview. Elevated heart rate during the latter, “recovery” phase was specific to C-PTSD, and was associated with higher subjective ratings of stress, shame, and guilt: in other words, it was associated with at least one component of DSO (32). This exaggerated and prolonged stress response may be linked to the DMN dysfunction seen in the prior study, as the DMN is normally activated during acute stress (33), but this needs to be verified through simultaneous investigation of central and peripheral responses to trauma-related cues. Studies examining physiological markers of dissociation in PTSD, which may be relevant to C-PTSD, have failed to yield consistent results (34).

A single study has examined epigenetic modifications in elderly individuals with C-PTSD. In this sample, altered DNA methylation was observed in the genes *HAP1*, *RANBP2* and *PSMA4*. These genes have been tentatively linked to neural processes such as neurogenesis, inhibitory neurotransmission, and memory formation, but their exact significance in this context is unknown (35).

It is possible that further refinements in neuroimaging research, or in combinations of neuroimaging and peripheral stress markers, may provide reliable biomarkers of C-PTSD (36). However, at this moment, no anatomical or physiological change consistently linked to the unique symptoms of C-PTSD has been identified. Where might such specific biomarkers be found? Surprisingly, there are several promising leads from animal models of prolonged traumatic stress.

## Animal models of C-PTSD

At first sight, it may seem difficult, if not impossible, for an animal model to replicate the features of PTSD, particularly those of the DSO domain, which require high levels of conscious awareness and cognition (37). Nevertheless, evidence of a C-PTSD-like phenotype has been observed in several mammalian species, including rodents, equines, and primates. In these species, prolonged or repeated trauma has been associated with impairments in emotional responses and social behavior which resemble two of the three domains of DSO (38–41). For example, in prairie voles, which exhibit monogamous behavior similar to that seen in humans in the wild, prolonged stress led to impaired pair bonding with female partners, and “indiscriminate huddling” with other female voles (41). Such animals also exhibit features of PTSD such as increased vigilance and exaggerated startle responses. These changes have been associated with a wide range of chronic trauma exposures, including experimental repeated stress, captivity, forced work, or maltreatment by humans (39, 40).

Based on these results, rodent studies have been carried out to examine the physiological and biochemical correlates of exposure to chronic or recurrent trauma. Experimental stressors used to induce a “C-PTSD-like” phenotype in these animals include predator scent stress (PSS), stress-restress, and combinations of more than one type of stressor. Peripherally, chronic traumatic stress is associated with reduced serum and adrenal cortisol and altered adrenocortical histology, which correlates with levels of observed anxiety (42). Centrally, exposure to recurrent traumatic stress has been associated with increased cerebellar noradrenaline levels, reduced levels of dopamine in the brainstem, hypothalamus and hippocampus, and increased hypothalamic corticotrophin-releasing hormone (CRH) (43, 44). In addition, chronic trauma appears to be associated with increased expression of the neuropeptide vasopressin (AVP) in the paraventricular nucleus (PVN) of the hypothalamus, and more specifically in the magnocellular portion of the PVN (44–46). Such trauma is also associated with reduced oxytocin expression in the nearby supraoptic nucleus (SON), which correlates with impaired pair-bonding behavior (41). While alterations in monoamine transmitters and HPA axis hormones have been observed in non-complex PTSD, alterations in AVP and oxytocin may be particularly relevant to C-PTSD, as they may represent an evolutionary “bridge” between animal and human phenotypes of this condition.

As C-PTSD is often linked to childhood abuse or neglect, animal models of childhood adversity, may also be used to approximate this disorder in mammals. Rats exposed to early maternal separation show evidence of increased fear conditioning and impaired social behavior, which are consistent with the two dimensions of C-PTSD. These changes are associated with blunted vasopressin release, reduced expression of the neurotensin 1 receptor, and overexpression of the chloride channel NKCC1 (47–50). Moreover, these changes may be reversible through the administration of oxytocin (49, 50). These results are broadly consistent with those seen in animals with other forms of chronic trauma. Nevertheless, these findings should be interpreted with caution, because early childhood adversity is associated with a wide range of psychiatric disorders besides C-PTSD (47).

## An evolutionary perspective on C-PTSD

Viewed at a surface level, the behaviors exhibited by persons with C-PTSD seem grossly maladaptive. For example, why do many of those affected by this disorder avoid help-seeking, idealize their abusers (the so-called “Stockholm syndrome”), or enter subsequent relationships where there is a high risk of experiencing abuse? (2, 51) Such paradoxical phenomena may be explicable in terms of the appeasement displays seen in animals, which lead to a “conditional reconciliation” between aggressor and victim and ensure individual survival and well-being. For example, in a fight between male chimpanzees, appeasement of the defeated or “subordinate” male protects it against death or mutilation, and ensures its survival in the larger group. In situations characterized by traumatic “entrapment,” where the victim cannot easily “escape” from the abuser, similar behaviors may become established in humans and dominate over more “adaptive” strategies such as seeking help outside the immediate social circle. Such situations include intimate partner violence (IPV) and childhood physical and sexual abuse, both of which are strongly associated with C-PTSD. In the words of Cantor and Price (2007), “appeasement is the most likely endophenotype for complex PTSD.” (52) The exact forms that these appeasement displays may take depend on interactions between evolutionarily primitive (hypothalamic and brain stem) brain structures that mediate a general, undifferentiated appeasement response, and more recently evolved (limbic and cortical) structures that modify its expression and lead to a disturbance of self-concept (52, 53). Such a model is attractive because it provides a plausible explanation for the DSO dimension of C-PTSD: many aspects of this dimension, such as shame, dissociative symptoms, and submissiveness towards aggressors, can be understood as variations of appeasement (54).

If the above hypothesis is true, then biological alterations associated with appeasement or subordinate status in animals may be implicated in the pathogenesis of C-PTSD, and even serve as biomarkers. In this context, it is helpful to briefly review what is known about the biological correlates of these behaviors. In mammals exhibiting cooperative behavior, acute defeat in a conflict is associated with increased cortisol, whereas a more stable subordinate status is associated with lower levels of cortisol than those seen in dominant animals (55). Similar changes have been observed in male capuchin monkeys, where dominant males had elevated levels of testosterone and cortisol compared to subordinates (56). In female rhesus monkeys, appeasement behavior is inversely correlated with 5-HT_1A_ binding potential in the OFC, and a functional polymorphism (short or *s* variant) of the 5-HT transporter gene *SLC6A4* was linked to a decrease in appeasement behavior with age (57).

Neural correlates of subordinate social status have also been studied in non-mammalian species. In the cichlid fish species *Neolamprologus pulcher*, subordinate fish had higher levels of arginine vasotocin, the analogue of AVP. In addition, levels of isotocin, the analogue of oxytocin, were negatively correlated with affiliative social behavior (58). In the electric fish *Gymnotus omarorum*, alterations in hypothalamic vasotocin are also associated with the establishment of dominant-subordinate social hierarchies (59). These findings are consistent with research in rodents, which have also found associations between AVP and oxytocin receptor densities in the limbic system and hypothalamus and social dominant/subordinate status (60, 61).

A tentative synthesis of these findings is that C-PTSD-like phenotypes in animals, whether in the wild or in experimental settings, are associated with alterations in serotonergic transmission, HPA axis function, and dysregulation of the similar neuropeptide transmitters oxytocin and AVP. The latter two are particularly significant because they play a central role in social behavior in both animal humans, including affiliation, social bonding, and appeasement and submission displays (62, 63). Alterations in these peptide transmitters are likely to be associated with the DSO symptoms that are specific to C-PTSD, and more particularly with disturbances in interpersonal and social relationships (41). Such alterations may reflect a dysregulation in evolutionarily ancient neurochemical processes mediating submission and appeasement displays, triggered by exposure to prolonged trauma in an interpersonal setting (51).

If C-PTSD reflects a dysregulated or morbid form of appeasement behavior, does this arise from chronic trauma, from antecedent vulnerability factors, or from both? Research on the epidemiology of C-PTSD has shown that it is more prevalent in those who occupy a “subordinate” position in human social hierarchies – women, migrants, persons abandoned by their parents in childhood, and those with a lower socioeconomic or educational status (64–66). Animal studies of early maternal separation also show evidence of altered neuropeptide signaling and deficits in social behavior, even if they do not exhibit features of PTSD (47, 50). Together, these findings suggest that C-PTSD may result from the superimposition of PTSD on a prior state of deficits in social organization, which result from psychosocial disadvantage and are further exacerbated with the onset of PTSD symptoms. This hypothesis is entirely consistent with the available evidence, but it requires verification through the assessment of DSO symptoms – and their biological correlates – in children and adults belonging to “subordinate” social groups, both with and without C-PTSD (52, 64).

## Summary and implications for the treatment of C-PTSD

**Figure 1:**
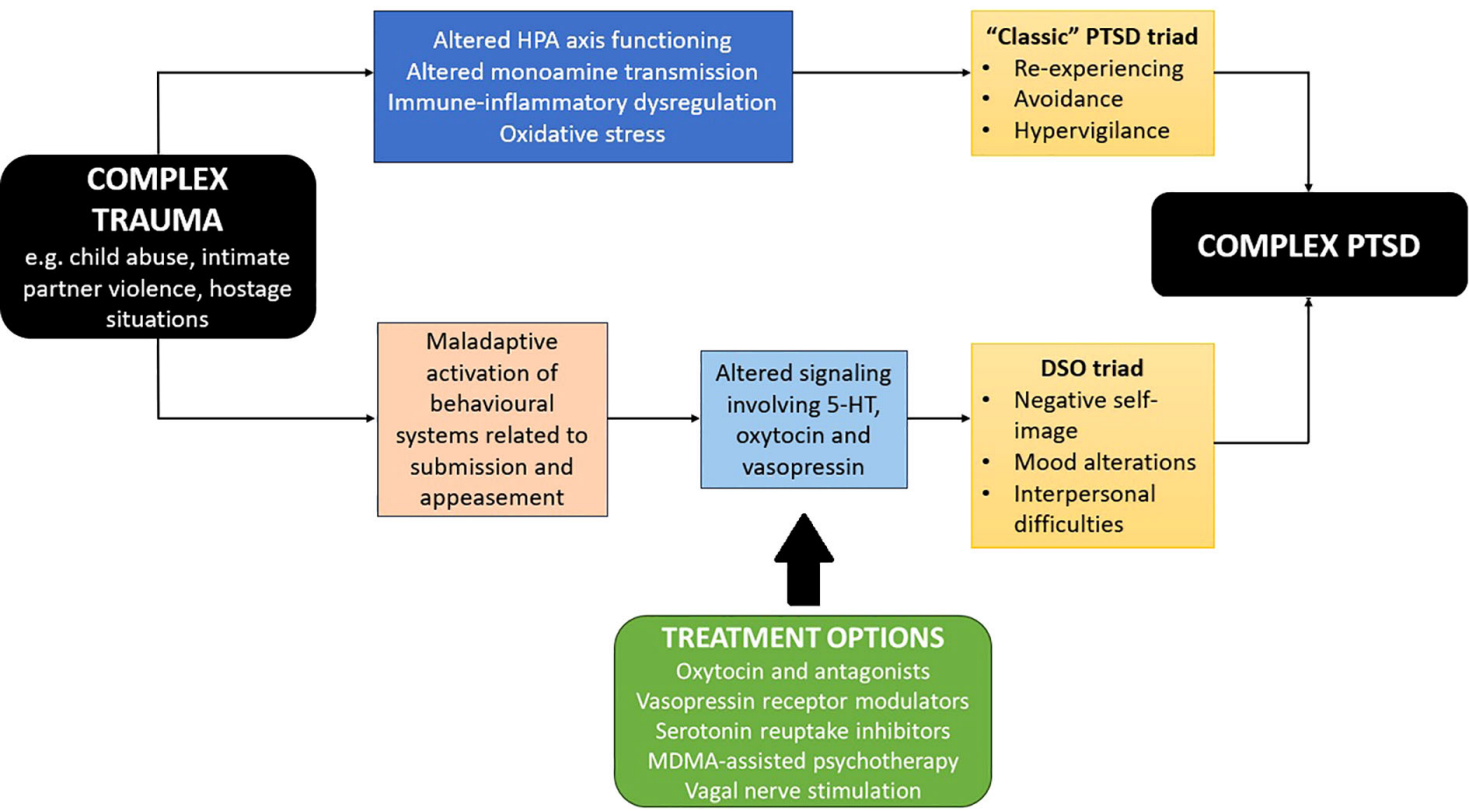
Pathophysiology of complex PTSD based on human research and animal models, including evolutionary theory. 5-HT, 5-hydroxytryptamine (serotonin); DSO, disturbances in self-organization; HPA, hypothalamic-pituitary- adrenal (axis); MDMA, 3,4-methylenedioxymethamphetamine; PTSD, post-traumatic stress disorder.

A tentative synthesis of animal and human research into C-PTSD, indicating possible points of converge and their “deep” evolutionary roots, is presented in **Figure 1**. In this model, complex trauma activates both the mechanisms responsible for PTSD symptoms, and a behavioral system involved in appeasement and submission displays in the face of threats to one’s survival. The latter system may have been sensitized by prior psychosocial adversity or disadvantage. Dysregulation of this system leads to the DSO symptoms that characterize C-PTSD. Such dysregulation may involve altered expression of the genes identified in human research on C-PTSD. For example, the *HAP1* gene codes for huntingtin-associated protein 1, which helps maintain the integrity and connectivity of 5-HT neurons (67). Altered expression of this protein may alter serotonergic transmission, leading to dysfunctional appeasement behavior.

The conclusions advanced above are tentative, and need to be verified in human subjects. Research on alterations in oxytocin and AVP in patients with PTSD have yielded mixed results, but none of these studies have specifically examined C-PTSD: most of these were conducted in veterans with acute trauma related to combat situations (68–72). Genetic variants in oxytocin and vasopressin receptors (*OXTR* and *AVP1a*) appear interact with mother-child attachment to influence vulnerability to PTSD in children exposed to an armed conflict. Such a finding may be of relevance to the genesis of C-PTSD, which often occurs in relation to parental neglect and abuse (73).

The above model also has implications for effective treatment of the DSO symptom domain in C-PTSD. Clinical trials of agents acting at AVP or oxytocin receptors in PTSD have also yielded conflicting but suggestive findings. A controlled clinical trial found that balovaptan, an antagonist of the AVP1a receptor, did not differ from placebo in reducing PTSD symptoms, but this study did not include subjects with C-PTSD (74). On the other hand, administration of a single dose of intranasal AVP to 12 persons with PTSD led to increased responsiveness when their spouse or partner expressed anger. This suggests that AVP influences social behavior in this population (75). Similarly, trials of intranasal oxytocin in PTSD have found that this drug may improve social cognition and emotion recognition (76, 77). It is plausible that AVP or oxytocin, or synthetic agonists activating their receptors, may reduce the interpersonal deficits seen in C-PTSD, though this remains to be verified.

As noted above, animal models have identified a possible interaction between 5-HT and these peptides in studies of social hierarchy. Administration of the serotonin reuptake inhibitor (SRI) paroxetine appears to reverse the social deficits caused by prolonged stress (41), and social status is associated with altered 5-HT_1A_ receptor density in the midbrain and hypothalamus (61). SRIs are approved pharmacological treatments for PTSD, and improve social functioning over a period of two years, though less reliably than psychological interventions (78). A trial examining patients with PTSD exclusively caused by trauma in an interpersonal setting (i.e., physical or sexual abuse in childhood or adult life) found that the SRI sertraline was superior to placebo; this result is significant because C-PTSD commonly results from interpersonal trauma (79). More recently, 3,4-methylenedioxymethamphetamine (MDMA), which increases 5-HT release, acts as an agonist at 5-HT_2A_ receptors, and inhibits 5-HT reuptake, has been found to improve DSO symptoms such as impaired self-image and mood instability when used as an adjunct to psychotherapy in PTSD (80). In healthy adults, MDMA has positive effects on social behavior (81) and acutely increases oxytocin and vasopressin release (82, 83). This drug may represent a potential breakthrough in the management of C-PTSD, but it is a controlled substance with a high potential for misuse and a narrow therapeutic index. It may be possible to reduce these risks by using isomers of MDMA, as they differ in their pharmacological properties: S-MDMA increases serotonin and oxytocin release, while R-MDMA acts predominantly at 5-HT_2A_ receptors and has “psychedelic” properties (82).

Apart from these treatments, research in an animal model of C-PTSD has found that vagus nerve stimulation (VNS) leads to symptomatic improvement (84). In humans, VNS is used primarily to treat resistant major depression, though it has also proved helpful in PTSD resistant to standard treatments (85). The benefits of VNS in PTSD appear to correlate with changes in cortical glutamatergic transmission (86), but VNS has effects on multiple neurotransmitters, including serotonin (87) and oxytocin (88). Due to this, it is possible that C-PTSD may respond to VNS where other treatments have failed. In another animal model of C-PTSD, the use of LK00764, an experimental agonist of trace amine-associated receptor 1 (TAAR1), prevented the emergence of behavioral problems following predator stress. This was associated with reduced levels of 5-HT in the hippocampus and dopamine in the corpus striatum (89). Modulation of TAAR1 may represent a future avenue for beneficial modulation of 5-HT alterations in patients with C-PTSD.

The “biological” findings mentioned above are also relevant to the psychosocial treatment of C-PTSD. If DSO symptoms are a biological reflect trauma and ongoing psychosocial disadvantage, psychological interventions should focus not only on the traumatic situation, but on ameliorating social and economic hardship and ensuring patient safety (3, 7, 8). In children, childhood neglect is associated with both environmental under-stimulation and C-PTSD, which can contribute to DSO. Animal research has shown that environmental enrichment can reverse the biobehavioral effects of early maternal loss, and this may be a useful strategy for treating C-PTSD in youth (50). In adults, similar benefits may be obtained through enhancing social support and the affected individual’s social orientation, particularly in persons belonging to socially marginalized communities (90). More recently, it has been suggested that C-PTSD may respond to interventions that aid in building resilience and fostering adaptive personality development. This process, known as “post-traumatic growth” (PTG), has been documented across a wide range of traumatic stressors, including life-threatening illnesses and natural disasters. Anecdotal evidence suggests that focusing on PTG, through the enhancement of social connection, and the use of cognitive-behavioral and psychodynamic therapy techniques, may improve outcomes in those suffering from C-PTSD (91). Little is known about the biological correlates of PTG, though it has been theorized that oxytocin can facilitate this process (92).

## Limitations

Certain caveats must be kept in mind when appraising the data cited above. First, research into the biological roots of C-PTSD is still in its early stages, and the conclusions reached above are likely to change as further data accumulates. Second, it is not yet clear how the neuroimaging findings in humans with C-PTSD can be aligned with the biochemical changes observed in animal models of this condition. Third, studies of C-PTSD in humans involve significant heterogeneity, both in terms of case definition and of the type of complex trauma involved. Fourth, the validity of animal models of C-PTSD has not yet been established robustly, particularly with regard to DSO symptoms. Finally, the link between appeasement or submission displays in animals and C-PTSD in humans, though consistent with the available evidence, remains a hypothesis. As more is learned about both the biology underlying these phenomena, this hypothesis may be either confirmed or refuted.

Technological advances may help overcome some of these concerns. Recent research has investigated the use of machine learning models in analyzing biomarker data in patients with PTSD. Though this work is still in its early stages, such models have been found useful in predicting PTSD based on clinical and biochemical parameters, and in identifying genes related to immune-inflammatory dysfunction in PTSD (93, 94). Such approaches may be fruitfully employed in future to identify patterns in clinical and biomarker data from patients with C-PTSD, with a specific focus on indices of peptidergic and serotonergic transmission, neuroimaging parameters, and neurophysiological data.

## Conclusions

Complex PTSD is a source of significant suffering and disability, and is often challenging to treat in clinical practice. Evidence from recent animal research suggests that the symptoms unique to this condition are related to alterations in neurotransmission involving serotonin, oxytocin, and vasopressin. Using the conceptual framework of evolutionary theory, these changes can be linked to evolved behavioral systems involved in appeasement and submission displays that ensure survival when a social organism is faced with a threat from either conspecifics or predators. Though this model requires further exploration, it serves as a source of testable hypotheses regarding dysregulation of these transmitters and their receptors in patients with C-PTSD, which could serve as reliable biomarkers of this condition or its symptom dimensions. Early evidence from human research suggests that pharmacological and neuromodulation strategies can have beneficial effects on these transmitter systems, reducing the disturbances of self-organization that are characteristic of C-PTSD. Such interventions should not be viewed in a reductionist manner. They probably work best as part of a holistic treatment approach that involves trauma-focused therapies and measures to ensure the sufferer’s safety and overall welfare, and foster post-traumatic growth and resilience.
